# ^19^F NMR viewed through two different lenses: ligand-observed and protein-observed ^19^F NMR applications for fragment-based drug discovery

**DOI:** 10.1039/d1cb00085c

**Published:** 2021-07-12

**Authors:** Caroline R. Buchholz, William C. K. Pomerantz

**Affiliations:** Department of Medicinal Chemistry, University of Minnesota 308 Harvard Street SE Minneapolis Minnesota 55455 USA wcp@umn.edu; Department of Chemistry, University of Minnesota 207 Pleasant St. SE Minneapolis Minnesota 55455 USA

## Abstract

^19^F NMR has emerged as a powerful tool in drug discovery, particularly in fragment-based screens. The favorable magnetic resonance properties of the fluorine-19 nucleus, the general absence of fluorine in biological settings, and its ready incorporation into both small molecules and biopolymers, has enabled multiple applications of ^19^F NMR using labeled small molecules and proteins in biophysical, biochemical, and cellular experiments. This review will cover developments in ligand-observed and protein-observed ^19^F NMR experiments tailored towards drug discovery with a focus on fragment screening. We also cover the key advances that have furthered the field in recent years, including quantitative, structural, and in-cell methodologies. Several case studies are described for each application to highlight areas for innovation and to further catalyze new NMR developments for using this versatile nucleus.

## Abstract 2

^19^F NMR has emerged as a powerful tool in drug discovery, particularly in fragment-based screens.

## Graphical abstract

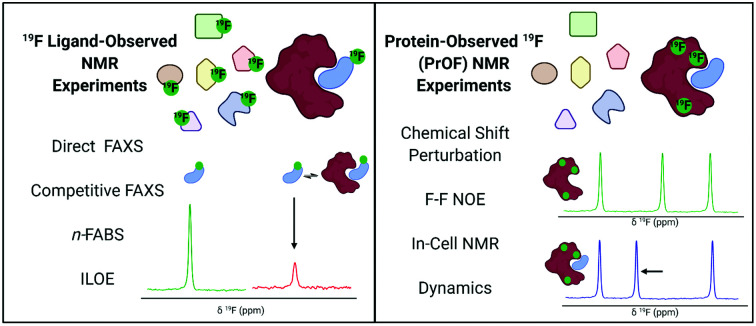

## 1. Introduction

### The influence of fluorine at the chemistry and biology interface

Innovations in using organofluorine compounds at the chemistry and biology interface have continued to emerge over the last 80 years. Developments in biomedicine, including early work on anesthetics,^1^ PET imaging,^2^ magnetic resonance imaging,^3^ nuclear magnetic resonance spectroscopy (NMR),^4^ blood substitutes,^3^ herbicides,^5^ and FDA approved drugs,^5^ support the broad utility of an atom that lacks sufficient endogenous levels in biological systems. With the highest electronegativity of all atoms, fluorine forms the strongest chemical bonds with carbon (*e.g.*, 131 kcal mol^−1^ C–F bond dissociation energy for CF_4_).^6^ Given the significant bond strength from fluorination, and low susceptibility to oxidation, carbon–fluorine bonds impart metabolic stability to biomedicines. However, early evidence of significant toxicity with low molecular weight fluorinated anesthetics, demonstrated that organofluorine compounds were not as physiologically inert as previously thought.^1^ We now know that in addition to providing a protective role for new medicines, organofluorine functional groups can engage in a variety of noncovalent interactions, including dipolar interactions and sometimes weak hydrogen bonds, as well as significantly altering the physicochemical properties of fluorinated molecules.^7–9^

In 2007 a comprehensive analysis by Müller *et al.* showed that ∼20% of all pharmaceuticals contained at least one fluorine atom.^5^ Although this statistic is often cited, this percentage has risen in recent years. While 29% in 2011,^10^ percentages of fluorinated small molecule drugs increased to 47% in 2018,^11^ 41% in 2019,^12^ and 33% in 2020.^13^ ††Imaging agents, peptides, nucleic acids, and supplements are excluded in these analyses. This rise can in part be attributed to both an increased knowledge of new strategies for using fluorine in biomedicine,^14^ new biomedical applications for organofluorine molecules,^3^ and a significant increase in synthetic methods for fluorination.^15^

Given that NMR has played a prominent role in the early stages of drug discovery, and the increased number of fluorinated small molecule drugs cited above, ^19^F NMR has emerged as an important tool for drug development. ^19^F NMR of small molecules (ligand-observed ^19^F NMR) preceded target-based biomolecular ^19^F NMR and as such is more developed.^4,16–18^ However, NMR applications of fluorinated proteins (protein-observed ^19^F NMR) is gaining increasing attention.^4,19–21^ Here we provide an update on new applications in ligand-observed and protein-observed fluorine NMR in drug discovery, with a particular focus on the emerging applications in fragment-based drug discovery (FBDD).

### A brief timeline for ^19^F NMR development

**Fig. 1:**
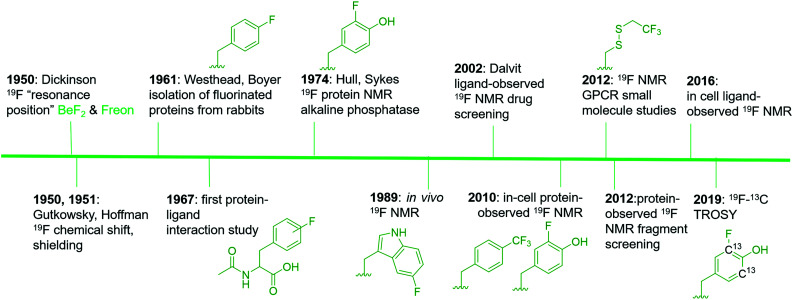
An abbreviated timeline of ^19^F NMR development with select examples.^22–24,27–31,34–38,148^ Fluorinated molecules shown in green. Side chains from left to right are: 4-fluorophenylalanine (4FF), 3-fluorotyrosine (3FY), 5-fluorotryptophan (5FW), trifluoromethyl-L-phenylalanine (tfmF), 3FY, 2,2,2-trifluoroethanethiol (TET), and ^13^C-labeled 3FY.

^19^F is a spin ½ nucleus and a 100% abundant isotope with a gyromagnetic ratio close to ^1^H NMR leading to an 83% similar signal sensitivity.^21^ Given these favorable magnetic resonance properties, ^19^F NMR studies of fluorinated molecules have provided foundational knowledge of magnetic resonance properties since the early stages of NMR research. Dickinson in 1950, provided some of the first characterization of chemical shifts using solutions of inorganic and organofluorine compounds (Fig. 1).^22^ Using a series of fluorinated molecules, Gutkowsky and Hoffman also provided early analyses of magnetic nuclear shielding and coupling for deducing effects on charge distribution and molecular structure.^23^ In the context of biomolecular ^19^F NMR, fluorinated phenylalanine derivatives were first used to study protein–ligand interactions with chymotrypsin in 1967.^24^

Access to fluorinated proteins for protein-observed ^19^F NMR was facilitated by prior studies interested in the plasticity of the biosynthetic machinery for tolerating unnatural amino acids. Fluorinated phenylalanine and tyrosine were first studied for their effects on bacteria and demonstrated a growth inhibition effect,^25^ which was overcome through sustained culturing.^26^ Boyer and Westhead subsequently showed 4-fluorophenylalanine could be incorporated into proteins when fed to rabbits, and the isolated proteins retained enzymatic activity.^27^ Ultimately, Hull and Sykes characterized the first fluorinated protein by ^19^F NMR, 84 kDa alkaline phosphatase in 1974, which was produced in *E. coli via* metabolic labeling with 3-fluorotyrosine.^28^

The first applications for drug screening were pioneered by Dalvit and co-workers who have since developed a suite of techniques for screening fluorinated ligands against proteins.^29–31^ The absence of fluorine in naturally occurring biomolecules, and the high sensitivity of the fluorine nucleus for detecting binding interactions, has led to significant interest in this approach over the last two decades in both academia and the pharmaceutical industry. The impact of this approach can be seen through the development of new pulse sequences, improved NMR probe designs, and commercial access to fluorinated small molecule libraries.

Protein-observed fluorine NMR while originally increasing the size of proteins that could be readily studied by biomolecular NMR, was largely limited to the study of protein folding and molecular recognition of native substrates.^32^ Concerns over the significant chemical shift anisotropy (CSA) leading to significantly broader resonances than fluorinated small molecules was one potential limitation for applying this approach to small molecule screening.^30,33^ Despite this concern, the first analysis of small molecule agonists and antagonists with the β-adrenergic receptor was reported by Wüthrich and co-workers in 2012.^34^ The same year, the fluorinated KIX domain of coactivator CBP, was used in a preliminary demonstration of small molecule screening to discover inhibitors of protein–protein interactions.^35^ The significant CSA challenge is beginning to be addressed through ^19^F–^13^C transverse relaxation-optimized spectroscopy (TROSY)-based NMR methods.^36^ Improved cryo-probe designs for increasing both the size-range of fluorinated proteins, the sensitivity of the NMR experiment, and the lack of a biological background also offer exciting opportunities for in-cell NMR.^37,38^

To date, both ligand-observed and protein-observed ^19^F NMR methods have become increasingly adopted in drug discovery applications with many opportunities for innovation. The next section will highlight some general principles to be considered for ^19^F NMR followed by specific discussions of ^19^F NMR in drug discovery.

### Spectral behavior of the ^19^F nucleus for ^19^F NMR

One significant advantage of ^19^F NMR over ^1^H NMR methods when used for small molecule discovery is its hyperreponsiveness to changes in chemical environment. While the traditional range of chemical shifts for ^1^H NMR spans ∼14 ppm, the fluorine chemical shift range has been reported to be up to 800 ppm wide.^39^ For fluorinated side chains found in proteins, this range approaches 170 ppm.^20^ This range is comparable to the ∼200 ppm range of chemical shifts for drug-like motifs.^40^ In the context of different chemical environments being produced upon small molecule binding, Urick *et al.* found ^19^F resonances from a fluorinated tryptophan to be ∼6–20 times more responsive than ^1^H resonances on the same amino acid.^41^ Even solvent isotope effects on the chemical shift from H_2_O and D_2_O can be observed for ^19^F resonances, leading to chemical shift differences up to 0.25 ppm.^42^ The solvent isotope experiment has been used as a sensitive method for characterizing solvent exposure of fluorinated protein side-chains.^43^

The chemical shift sensitivity difference between ^19^F NMR over ^1^H NMR can be attributed to differential contributions to the nuclear shielding parameters. The nuclear shielding parameter is composed of both diamagnetic and paramagnetic shielding contributions. Diamagnetic shielding is based on ground state contributions of the electrons in the presence of an external field, whereas the paramagnetic term is based on coupling of ground and excited state orbitals and is thus dependent on their energy difference.^44^ In the case of ^1^H chemical shifts, diamagnetic shielding is typically the main contribution dominated by electrons in the 1s orbital. However for ^19^F NMR, either term can dominate due to low lying orbitals from fluorine's lone pair electrons.

Despite the large chemical shift window for ^19^F NMR, significant progress has been made in quantum chemical method developments for accurate prediction of fluorine chemical shifts. This is particularly relevant for fluorinated small molecules which can be calculated using relatively low level theoretical methods as these molecules possess fewer atoms and dynamic conformations than fluorinated biopolymers.^45^ In the case of fluorinated proteins, Oldfield and co-workers were the first to successfully report accurate prediction of the fluorine chemical shifts of a 5-fluorotryptophan-labeled *E. coli* galactose binding protein which contains five tryptophans with fluorine resonances spanning ∼10 ppm.^46^ They concluded that weak and long-range electronic interactions were the dominant contribution to the chemical shift dispersion *versus* van der Waals effects. Conversely, Lau and Gerig's analysis of a more dynamic protein, dihydrofolate reductase, was unable to effectively account for all aspects of nuclear shielding contributions and dynamic motions to accurately predict chemical shifts.^47^ More recent efforts using quantum chemical methods by Isley *et al.* studying 3-fluorotyrosine-labeled BRD4, highlight the importance of accounting for explicit solvation of the fluorinated amino acids which can significantly affect chemical shift predictions leading in some cases to significant error.^48^ Accurate prediction of fluorine chemical shifts in proteins still remains a significant challenge.

In the context of ligand binding experiments, chemical shift perturbations can be used for sensitive quantitative affinity measurements; however, the limit of detection is based on both the rate of chemical exchange, as well as the relationship between the absolute affinity and the amount of receptor being used in the experiment. These experiments are discussed in subsequent sections.

### Chemical shift anisotropy effects on ^19^F NMR spectra

Transverse (*T*_2_) relaxation mechanisms significantly affect the observed line widths in ^19^F NMR spectra and are particularly sensitive to both the magnetic field strength and the size of the fluorinated molecule under study. For most organofluorine compounds, the predominant contributions to *T*_2_ relaxation are dipole–dipole interactions and CSA. For large fluorinated biomolecules, CSA-mediated relaxation is typically the dominant mechanism at high field strengths. Eqn (1) shows the *T*_2_ dependence on the square of the magnetic field strength (*ω*_F_), the shielding anisotropy of the nucleus (*σ*_‖_ − *σ*_⊥_), and the rotational correlation time (*τ*_C_) for the protein under study.^49^1
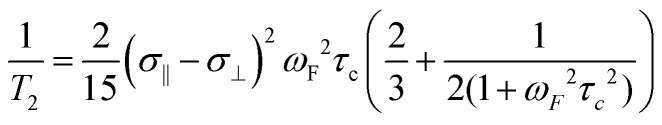


At 235 MHz Hull and Sykes showed approximately 50% of the line width of 3-fluorotyrosine-labeled alkaline phosphatase was caused by CSA, cancelling out the signal to noise and resolution gains from acquiring a spectrum at a 2.5 times higher magnetic field strength.^33^

To address the challenge of the large CSA effect, F–C TROSY methods have been developed for aryl ^19^F–^13^C bonds, using conditions where the dipole–dipole interactions and CSA cancel.^36^ This has led to the characterization of proteins as large as 180 kDa, and has more recently been applied to ^13^C-labeled fluorouracil in RNA including ligand binding studies.^50^ Conversely, CF_3_-TROSY may be limited to lower field strengths where the CSA effects are negligible. At high field strengths, TROSY effects were not observed due to the high CSA of the CF_3_ group.^51^ Given the significant effects on NMR spectra, the CSA effect and the magnetic field strength for acquiring the spectra, should be a significant consideration when choosing a ^19^F NMR experiment. This is particularly relevant for large biomolecules.

### ^19^F NMR applications in fragment-based drug discovery

**Fig. 2:**
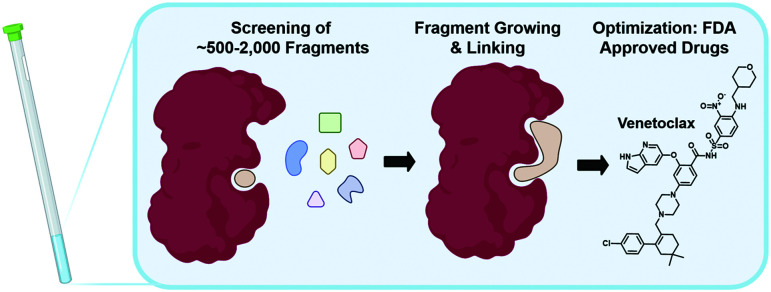
NMR in fragment-based drug discovery. Screening of libraries of ∼500–2000 fragments by NMR can be followed up by fragment growing and/or linking, and finally optimization to FDA approved drugs as in the case of Venetoclax.

The following sections will elaborate on the use of ^19^F NMR in the field of FBDD. The start of NMR research in this field began with the contributions from Abbott labs describing a structure–activity relationship (SAR) by NMR approach in 1996 using protein-observed methods.^52^^19^F NMR would subsequently be used as a complementary tool in 2002 focusing on ligand-observed NMR.^53^ The successful outcome of FBDD programs is supported by four FDA-approved drugs, with one originating from NMR screening (Fig. 2).^54–57^ FBDD offers a powerful solution to the chemical space challenge surrounding drug-like molecules of an approximate molecular weight up to 500 g mol^−1^, which is estimated to be ∼10^63^.^58^ FBDD approaches reduce this chemical space *via* screening low molecular weight molecules typically less than 300 g mol^−1^ to more effectively sample the chemical space necessary to bind a given target.^59^ This reduces the library sizes that need to be screened from tens of thousands of molecules to more manageable sizes typically ranging from 500 to 2000 compounds. The challenge associated with screening fragments is their low affinity for their target, typically in the micromolar to millimolar affinity range. As such, NMR is well-positioned as a sensitive biophysical technique to both detect and quantify these interactions. In the sections below, we will first discuss ligand-observed ^19^F NMR methods followed by a discussion of newer ligand discovery applications of protein-observed ^19^F NMR. For earlier examples, readers are referred to several prior reviews on this subject.^19,60,61^

## 2. Ligand-observed ^19^F NMR

**Fig. 3:**
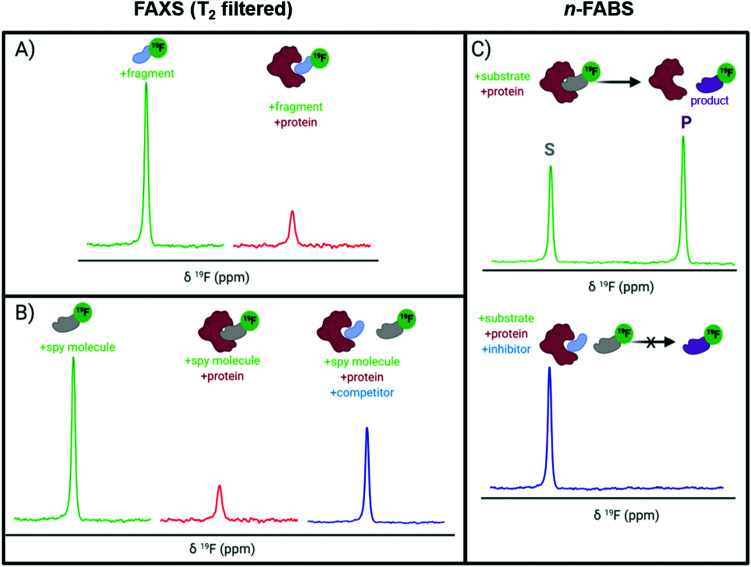
Idealized ^19^F NMR spectra for ligand-observed fragment screening. (A) Direct binding FAXS and (B) competitive inhibition FAXS using a CPMG pulse sequence where the resonance observed is from the free ligand in fast chemical exchange with the protein complex shown; and (C) *n*-FABS.

A key application of ligand-observed ^19^F NMR for small molecule screening takes advantage of the NMR properties of the biomolecular target that can be transferred to a small molecule in chemical exchange with the bound and unbound states. Based on the most commonly used approaches, ligand-observed ^19^F NMR experiments can be broadly broken down into direct binding fluorine chemical shift anisotropy and exchange for screening (FAXS) experiments, competitive inhibition FAXS, and *n*-fluorine atoms for biochemical screening (*n*-FABS), which is more specifically substrate/cofactor-based (Fig. 3). FAXS can be further categorized by the NMR parameter detected, but most commonly uses transverse relaxation filters. Other NMR experiments that have been described monitor changes in chemical shift perturbation and transferred or direct NOEs.^62^ These experiments have been prominently used for FBDD, and as such are extensively reviewed.^4,16–18^ In recent years several enabling methodologies have been developed that further the field, including creation of unique fluorinated fragment libraries,^63^ broadband protocols for expanded mixtures,^64^ quantitative methodology for measuring dissociation constants,^65,66^ multi-dimensional NMR experiments (*e.g.*, COSY,^67^ ILOE^62^), and screening protocols with both cellular lysates and intact cells.^68,69^ Many of these advances will be highlighted here.

### The *T*_2_ filter experiment

*T*_2_ relaxation filter experiments, like those used in FAXS, use a Carr–Purcell–Meiboom–Gill (CPMG) pulse sequence to detect ligand binding. In a CPMG pulse sequence a hard 90° excitation pulse is followed by a series of 180° refocusing pulses that create an echo.^70,71^ This echo decays exponentially as a function of *T*_2_ relaxation. When a ligand binds to a protein, the protein transfers its shorter *T*_2_, which is inversely related to molecular weight and magnetic field strength, and the echo diminishes faster in comparison to the free ligand. In practice, a *T*_2_-based filter experiment leads to detection of ligand binding events through the observation of a decrease in resonance intensity corresponding to the free ligand. This decrease in intensity is due to filtering out of the bound state contribution from the ligand that is in rapid chemical exchange. The large ^19^F CSA is particularly well-suited for *T*_2_ relaxation experiments. CSA is the main mechanism of *T*_2_ relaxation under high fields (>300 MHz)^72^ that are used today which can lead to large effects in signal perturbation from binding. A main limitation of CPMG experiments is the lack of a strong response for inhibitors binding in slow chemical exchange, a characteristic of high affinity interactions (*i.e.*, *K*_d_ < 1 μM). When screening low molecular weight fragments, this is usually less of a concern due to their typical weaker affinity and thus fast chemical exchange binding characteristics.

### FAXS in fragment screening

FAXS was first introduced by Dalvit *et al.* as a competitive binding assay for which a known fluorinated weak affinity binder, known as a spy molecule, is displaced by a competitive ligand.^31^ The binding event is monitored using a *T*_2_ filter-based CPMG pulse sequence. To identify a suitable spy molecule, a library of fluorinated molecules can be first screened against the target. Alternatively, a second approach can be to fluorinate a known ligand. An appropriate spy molecule will have a significant drop in signal when a CPMG pulse sequence is applied and protein is added which will create a large signal window for displacement by a competitive molecule. As a rule of thumb, a p*K*_d_ of 3.0–5.5 can lead to fast enough exchange kinetics to observe displacement over a wide affinity range while possessing sufficient affinity to form a protein complex with a significant population to detect changes in relaxation.^73^ The spy molecule can then be used to screen for new competitive inhibitors, both qualitatively through rank ordering inhibitors based on a return in resonance intensity, or quantitatively through inhibitor titrations. The latter experiments lead to the inhibition dissociation constant, *K*_i_, if the spy molecule's affinity (*K*_d_) has been previously determined using eqn (2) where *F* is the displacement value of the spy molecule upon addition of a competitive inhibitor.^30^2
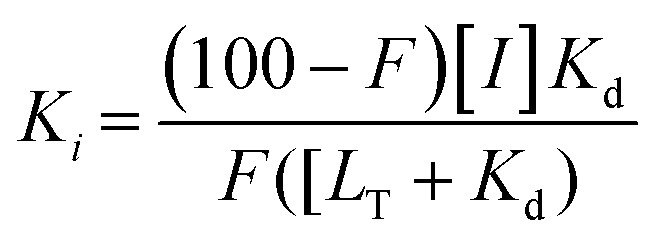


An attractive feature of this approach is that while spy molecules are weak affinity binders, they can be used to determine the *K*_i_ of high affinity ligands presuming sufficient protein is used to determine an accurate displacement value.^73^ This method is also suitable for fragment-based screening because of its ability to detect low affinity molecules while at low ligand concentrations which avoids solubility issues from screening ligands at high concentrations, typical in many biophysical fragment screening methods. This approach is also relatively high throughput due to the low amounts of protein required along with rapid affinity ranking and mixture screening. An advantage of the competitive mode is that a fluorinated library is not needed; however if fragment mixtures are used, hit mixtures will need to be deconvoluted.

A recent application of competitive FAXS in fragment-based drug discovery was a screen against sepiapterin reductase, a target in chronic pain.^74^ This assay was chosen because the competitive mode allowed the researchers to focus their screen on a substrate binding pocket, and detection of the ^19^F signal allowed for the use of large amounts of cosubstrate, NADP/NADPH. *N*-Acetylserotonin, a known binder (*K*_d_ ∼ 5 μM) was fluorinated with a CF_3_ moiety, to serve as the spy molecule. A return of signal intensity was readily observed upon spy molecule displacement by a known inhibitor used as their control. A 4,750-member library containing a diverse array of planar, sp^3^-enriched, and polar fragments was screened in mixtures of 12 using a CPMG pulse sequence. Out of 26 primary hits, 21 were confirmed in an enzymatic inhibition assay. Crystal structures and structure-based design led to a promising inhibitor with double-digit nanomolar potency, good ligand efficiency (LE, 0.53), and favourable absorption, distribution, metabolism, and excretion properties for further lead optimization.

FAXS screening using a fluorinated fragment library in the direct binding format can be another way to generate spy molecules without the need for subsequent chemical modifications of the hits. Furthermore, the direct mode can be used to screen for fluorinated inhibitor motifs since ^19^F is prevalent in drugs and can be an important functional group for non-covalent interactions.^75^ One emerging application for fragment-based ^19^F NMR screening is directed at nucleic acid drug targets. The first instance of ^19^F NMR fragment-based screening of a nucleic acid target was against telomeric repeat containing RNA (TERRA), an anti-cancer drug target because of the dependence on TERRA to form telomere heterochromatin in cancer.^76^ Known ligands of the G-quadruplexes that form TERRA *in vivo* were polyaromatic, making them poor candidates for drug discovery. To discover diverse, more drug-like chemical matter, a 355-member fluorinated fragment library with fragments containing either a CF_3_ or CF moiety was screened by FAXS in mixtures of eight. ^19^F NMR spectra were recorded, 1D-spectra with and without a CPMG *T*_2_ filter, for the fragment cocktails alone and in a mixture with TERRA_16_. A decrease in signal intensity and line broadening upon addition of TERRA_16_ in the *T*_2_ filtered spectrum was seen for 20 molecules (5.6% hit rate). Follow-up on seven of their hits demonstrated selectivity against tRNA and duplex DNA, indicating the sensitivity of this method for finding selective binders of noncoding RNA.

**Fig. 4:**
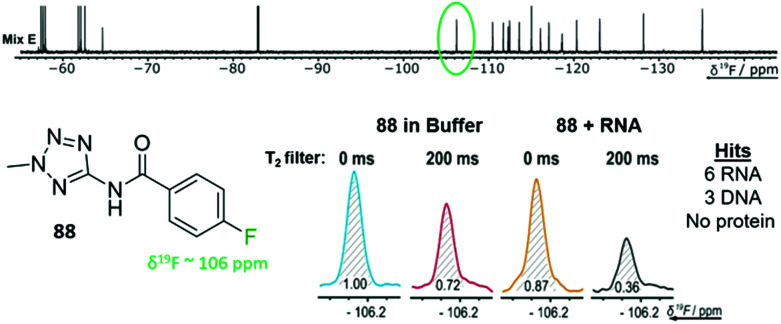
*T*_2_ filtered experiment against 24 different biomolecular targets. Example fragment **88** from fragment mixture E (top) and the corresponding ^19^F resonance of **88** with an RNA target (bottom). **88** was a hit for 9 of the 24 targets screened against. (Adapted from ref. 77 with permission from Wiley-VCH, copyright 2020).

FAXS screening has been further expanded to additional nucleic acid targets and takes advantage of the speed of the NMR experiments and the large mixture sizes that can be resolved. Enabled by the throughput of the NMR experiment, Binas *et al.* screened a fluorinated library against fourteen RNA targets while counter-screening against five DNA and five protein targets.^77^ 101 fluorinated fragments were screened against each target in five mixtures (20–21 fragments each) with and without a *T*_2_ filter (Fig. 4). Out of the 74 fragments that were classified as hits, proteins had the highest hit rate at 16–55 hits for each, excluding a phosphatase that has a known low druggability. RNA had the next highest hit rate, in particular riboswitch RNAs had 7–26 hits each. DNA had the lowest hit rates with the duplex only having one hit, and G-quadruplexes having 12–20 hits. There was some overlap in hits for each class of biomolecules (13 fragments hit all 3 classes), but there were also distinct hits for each class.

### *n*-FABS in fragment screening

*n*-FABS is an activity-based method where a substrate or cofactor of an enzymatic reaction is labeled with *n* fluorine atoms. In the original study, a CF_3_ moiety was appended onto the respective peptide substrate of the target proteins, AKT1 and trypsin, and the conversion of the enzymatically modified substrates to their respective products were detected by ^19^F NMR.^29^ This approach was advantageous for measuring IC_50_ values as one can detect both substrate and product. Additionally, the low nM amount of protein required makes it a useful method for rapid, high-throughput screening of fragments. Higher *n* values allow for even greater sensitivity.^78^ The primary drawback is the additional deconvolution experiments for mixtures that show activity, which uses additional reagents and time.

A recent example of *n*-FABS applied to fragment screening is against uridine nucleoside ribohydrolase (UNH), a target in *Trichomonas vaginalis* infection. Shea *et al.* had previously developed a ^19^F NMR activity-based assay following the conversion of 5-fluorouridine to 5-fluorouracil, a substrate and product that are already fluorinated, and screened the NIH clinical compound collection.^79^ After following up on several hits and concluding the target, UNH, was druggable, a fragment screen using a 2000-member library in mixtures of six fragments was employed to identify diverse chemical scaffolds that would be suitable for drug discovery efforts.^80^ Mixtures with at least 75% inhibition were deconvoluted and 97 hits were identified (4.9% hit rate). 18 compounds had an IC_50_ under 20 μM, several of which had a LE above 0.5, which is ideal for elaboration.^81^ In particular, a 3-hydroxypyrrolidine was further investigated as a fragment scaffold that could be expanded due to its potency, LE, and vectors for SAR exploration.^80^

### Fluorinated fragment libraries & efficient screening of chemical space

An essential component for ^19^F NMR fragment-based screening is the development of fluorinated fragment libraries, whether this be for spy molecule generation or direct screening. As this field has evolved, in-house collections, commercial libraries, and diversity-oriented synthesis methods have been developed. Single fluorinated motifs (CF, CF_2_, CF_3_), or motifs that can be decoupled are ideal when they result in isolated singlet ^19^F resonances,^40^ but polyfluorinated fragments are also used.^78,82–85^ Another consideration is the fraction of sp^3^ carbons (Fsp^3^) of the fragments. As inspiration, bioactive natural products tend to have more 3D-character. Recently a focus on 3D fragments and their importance has been studied and has resulted in the creation of Fsp^3^-rich libraries.^86,87^ Applying this to fluorinated fragments specifically, a recent report showed a diversity-oriented synthesis approach to create a more diverse fluorinated fragment library, the 3F library.^63^ 115 fragments were synthesized from nine core scaffolds and exhibited higher Fsp^3^ character and natural-product likeness, as well as ideal physicochemical properties, in comparison to two commercial libraries. To demonstrate the usability of this library a direct FAXS screen against four protein targets was employed. 105 of the fragments passed quality control and were screened in mixtures of 17–24 fragments with a *T*_2_ filter. Hit rates from 3–11% were obtained validating the utility of this diverse library in fluorinated fragment screening.

**Fig. 5:**
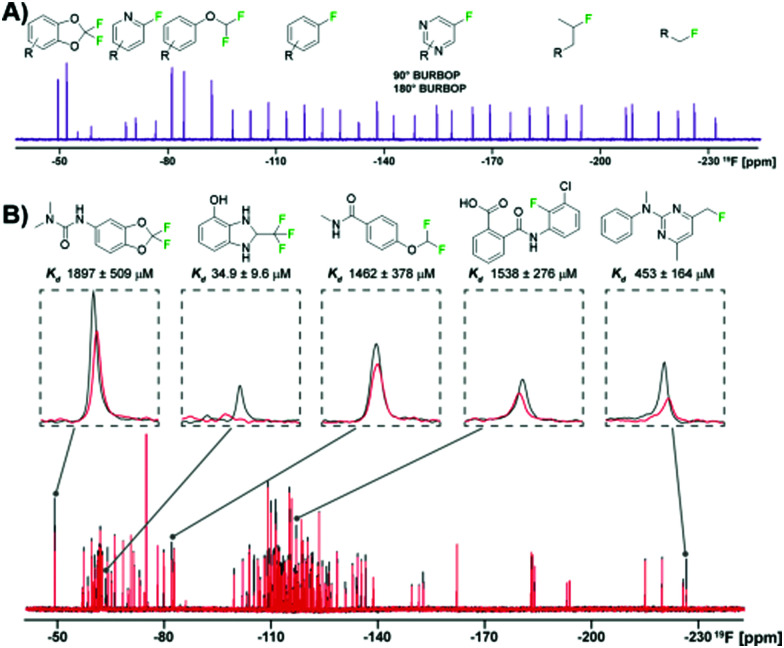
^19^F NMR broadband screening against CoaD. (A) ^19^F fragment mixture with diverse motifs covered by a BURBOP pulse in the resulting ^19^F CPMG experiment (adapted from ref. 64 with permission from Wiley-VCH, copyright 2020). (B) Representative screening hits from a supermixture of 152 fragments in absence (black) and presence (red) of the protein target CoaD (adapted from ref. 64 with permission from Wiley-VCH, copyright 2020).

To efficiently screen chemical space, it is necessary to balance screening a large number of fragments while also minimizing experiment time and reagents. One newly developed solution is to maximize the chemical shift range of detection to not only allow simultaneous screening of diverse fluorine motifs, but also to screen larger mixtures.^64^ The span of ^19^F drug-like motifs covers from −20 to −240 ppm.^40^ As described previously, current CPMG pulse sequences, the most commonly used ^19^F NMR experiment used in ligand-observed screening, consist of a hard 90° excitation pulse followed by 180° refocusing pulses. These limit the usable bandwidth to ±15 kHz to maintain 50% of the full signal integral. This means that mixtures have to be designed so that fragments only cover a small chemical shift range and several experiments must be performed to cover the whole range of drug-like motifs. Adiabatic 180° refocusing pulses, in the place of hard pulses, have previously been used to combat this issue, but only extend to ±30 kHz to maintain 50% of the full signal integral. To achieve a goal of at least ±60 kHz, which would cover most of the drug-like motif range, broadband universal rotation by optimized pulses (BURBOP), which are designed for nuclei with large chemical shift ranges, were developed into 90° and 180° pulses that are compatible with CPMG (Fig. 5a).^64^ Experimentally, it was shown to achieve on average 82% signal integral over 120 kHz. As an initial test, 35 compounds that spanned a chemical shift range of 183 ppm were sampled in a single 1D ^19^F CPMG experiment. In comparison, using hard pulses took four experiments and an adiabatic 180° pulse took two experiments to cover the whole range.

Demonstrating BURBOP applicability in screening, a new library, LEF4000, which is an expansion of the local environment of fluorine (LEF) library that focused on augmenting diversity based on the existing 2D fingerprint of fluorinated fragment library members, was screened against a bacterial enzyme target, CoaD.^64^ A hit rate of 6%, with hits encompassing the entire drug-like motif chemical shift range, demonstrated the importance of broadband methods such as this one that would allow efficient enrichment of existing fluorinated fragment libraries. Of these hits, the number of library members within a specific chemical shift range correlated with the number of observed hits, supporting a lack of bias towards a particular fluorinated motif. To test the applicability of “supermixtures,” 152 compounds were combined and screened at once using 5 hits from their original screen (Fig. 5b). The resulting signal reduction of hits were comparable to the spectra seen in the original screen with smaller mixtures, suggesting competition at the same binding site had little impact on hit detection, and demonstrating the utility of larger mixtures in screening.

### Quantification of protein ligand interactions by ^19^F NMR

Once a library has been screened, the next step is to prioritize fragment hits for follow up studies; quantification methods are critical for this. Biophysical methods beyond NMR commonly used in drug discovery sometimes do not work as well with fragment screening because of low fragment affinities and solubility issues. For NMR methods, as described previously, using a spy molecule in a competitive FAXS experiment can lead to *K*_i_ determination. However, establishing a spy molecule that is suitable can sometimes be difficult and the *K*_d_ cannot be derived readily from direct FAXS experiments when measuring *T*_2_ filter, STD, or water LOGSY effects because the response is not proportional to affinity.^41,88^ For *T*_2_ filter experiments this is because in the intermediate to fast exchange regime that fragments exist in, the observed relaxation rate (*R*_2,obs_) is the weighted average of the relaxation rate in the free (*R*_2,f_) and bound state (*R*_2,b_) plus a term that accounts for line broadening, the exchange term (*R*_ex_). Both *R*_2,b_, which dipolar relaxation (*R*_DD_) and CSA relaxation (*R*_CSA_) contribute to, and *R*_ex_ are unknown values limiting one's ability to extract a *K*_d_. Additionally, both terms vary widely making relative ranking of ligand binders challenging.

3 *R*_2,obs_ = *p*_f_*R*_2,f_ + *p*_b_(*R*_DD_ + *R*_CSA_) + *R*_ex_

To address this challenge, a new NMR methodology, chemical shift anisotropy ranking (CSAR), removes all other sources of relaxation so that only CSA relaxation is contributing to the relaxation rate, which can accurately be summed using chemical shielding tensor calculations to determine a fraction bound, and from there a *K*_d_.^65^ This is achieved by a spin lock frequency (10 MHz) in a high field (*e.g.*, 16.4 T) to eliminate the exchange term. In the elaborated methodology two different magnetic fields strengths are used to subtract out the dipolar relaxation contribution leaving only CSA, but in the simplified FastCSAR the dipolar relaxation is treated as constant between the free and bound state. Trypsin was used as a model protein to rank known ligands using CSAR and FastCSAR, and compared to the spy method to show the validity of this approach. There are limitations to this approach, including calculating chemical shielding tensors. Broader use beyond trypsin awaits further validation. However, FastCSAR has the advantage of ranking hits from one ligand concentration in comparison to a titration.

Another reported quantification method using direct FAXS relies on a ligand titration to quantify *K*_d_.^66^ This methodology only works for molecules with low binding affinities and conditions where the total ligand concentration is approximately equal to the concentration of free ligand, *i.e.* using low protein concentration. To demonstrate with direct FAXS using a *T*_2_ filter, HSP90 was screened at 0.8 μM with three ligands at varying concentrations up to 160 μM to derive a *K*_d_ by plotting transverse relaxation rate with and without protein ((*R*_2_)_+E+L_ − (*R*_2_)_−E+L_0__) as a function of ligand concentration (eqn (4)). A similar quantification can be done based on longitudinal relaxation rates (*R*_1_).4
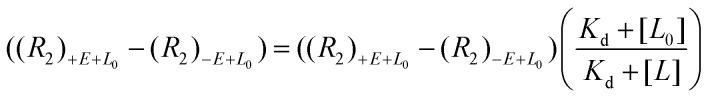


The utility of this approach is in the precise affinity ranking of hits from FBDD campaigns to select candidates for follow-up.

### ^19^F NOE for fragment linking

Fragment expansion is an important principle behind FBDD. Theoretically the binding energy of each fragment is additive when appropriately linked, and the entropic cost from binding two fragments is reduced creating a strong binder compared to the weak binding fragments on their own. However, this linkage is not trivial and can take trial and error to see improvements in affinity. A ^19^F NMR fragment-based screening approach against BACE-1, a drug target implicated in Alzheimer's disease, used ^19^F–^19^F intermolecular NOE to assist efficient linkage.^62^ First, a constructed fluorinated fragment library was screened and had a hit rate of 0.5%, which is comparable to traditional fragment screens against BACE-1.^89,90^ Hits were characterized and quantified by a differential chemical shift perturbation method, which was validated by parallel SPR *K*_d_ determinations. To pursue fragment linking, hits or seed fragments, were combined with other fluorinated fragments in ^19^F–^19^F NOESY experiments.^62^ The presence of ^19^F–^19^F NOEs indicated that fragments were binding simultaneously and were close in space (within ∼5 Å) and could potentially be linked. In a proof of concept, two protonated forms of fluorinated fragments were joined to form a compound with 100-fold potency increase relative to the original hit with a final IC_50_ of approximately 74 nM.^91^ This method is analogous to interligand NOE (ILOE) in ^1^H NMR. In later BACE-1 studies, a ^19^F NMR fragment screening campaign for a second site was completed followed by ^1^H–^1^H ILOE NMR experiments and molecular modeling to extend a lead compound into an additional pocket yielding a highly potent and selective inhibitor.^92^

### In-cell NMR screening by *n*-FABS

**Fig. 6:**
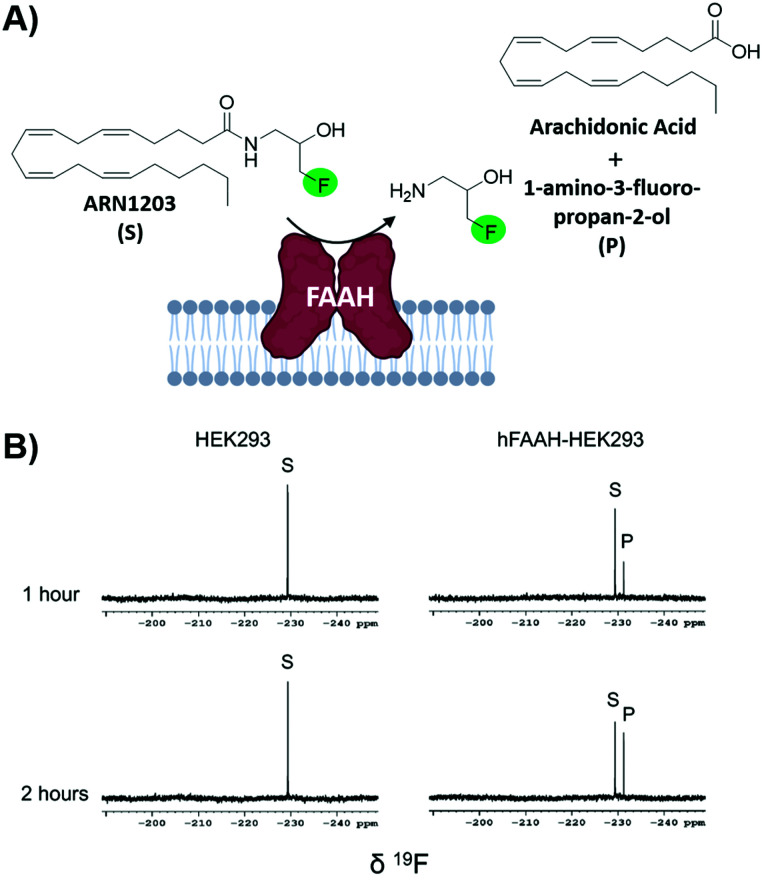
In-cell *n*-FABS assay for FAAH. (A) *n*-FABS assay setup for FAAH where a fluorinated natural substrate analogue, ARN1203, is cleaved in the presence of hFAAH to a traceable fluorinated product (B) ^19^F NMR spectra of intact HEK293 cells (control) and intact transfected HEK293 cells with ARN1203 (adapted from ref. 18 with permission from Springer, copyright 2020).

Fragment-based screening largely take place with isolated biomolecule targets. However, testing ligands closer to their true physicological conditions is desirable. New methodologies that permit screening in lysates and in living cells is valuable; the biorthogonality of ^19^F is well-suited for this type of screen. A new application using *n*-FABS has achieved this.^18^ In short, fatty acid amide hydrolase (FAAH), a target in pain and inflammation, was suitable for *n*-FABS screening because the lower enzyme quantities required could potentially overcome the challenges associated with screening against membrane-bound proteins like FAAH. A truncated form of FAAH fused to MBP that retained enzymatic activity but did not aggregate was screened using a fluorinated natural substrate analog, ARN1203 (Fig. 6a). Using an in-house generated library, 115 fluorinated fragments in mixtures of five were screened which led to a hit rate of 16.5%.^93^ This system was then taken to cell lysates using HEK293 cells that overexpressed human FAAH (hFAAH). Recombinant hFAAH expresses in low yield so screening in cell extracts is desirable. To validate the established 1-FABS assay using ARN1203 in lysates, a known inhibitor was used to quench the reaction. The determined IC_50_ for hFAAF lysate and the previously isolated MBP-rFAAH were compared for two known potent inhibitiors and two of the weaker binding fragments hits and found to be comparable.^69^ Following this success, the 1-FABS assay was taken into transfected HEK293 cells. Control experiments with ^19^F NMR spectra of cell lysate and supernatant showed that ARN1203 could get into cells, and that the product was found in the transfected but not control cells (Fig. 6b).^68^ The same potent inhibitors were used and shown to have comparable inhibition as in the cell lysate and fragments were used to show a dose–response demonstrating the sensitivity of the assay in cells.

## 3. Protein-observed ^19^F NMR

**Fig. 7:**
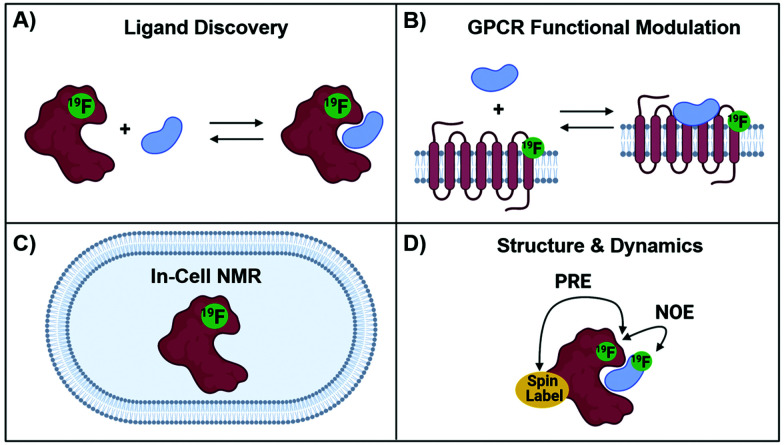
Protein-observed ^19^F NMR Studies. (A) Ligand discovery with soluble proteins (B) GPCR functional modulation with agonists and antagonists, (C) in-cell NMR and (D) structural and dynamics using NOE and PRE NMR.

The development of protein-observed ^19^F (PrOF) NMR applications for ligand screening was initially slower to develop than ligand-observed methods, but has since benefitted from the introduction of more sensitive cryoprobes to facilitate ligand screening. PrOF NMR takes advantage of perturbations in ^19^F-labelled protein resonances induced upon ligand binding. Proteins have been labelled with ^19^F to study protein folding and function by NMR since the seminal reports by Hull and Sykes in 1974.^28,60^ However, it is only in the past decade that PrOF NMR methods have been applied to small molecule discovery and screening efforts. A strength of PrOF NMR in studying protein–ligand interactions, particularly in the area of FBDD, is the rapid determination of a *K*_d_ for weak binding ligands while providing structural information regarding the ligand binding site and allosteric effects. Methods for incorporating fluorine into proteins have been previously reviewed,^94,95^ but include amber suppression,^96,97^ post-translational bioconjugation with reactive fluorinated molecules,^98,99^ enzymatic labeling,^100^ and metabolic labeling.^94^ Given the relatively conservative replacement of a hydrogen to fluorine atom, many different fluorinated amino acids are recognized by the natural biosynthetic machinery. This has led to metabolic labeling to be the most commonly used form of protein labeling, with over 21 different amino acids being reported in the literature.^20^ As PrOF NMR has become more widely adopted, proteins as large as 180 kDa have been studied by this approach,^36^ and have included both soluble and membrane-bound proteins, as well as multidomain proteins (Fig. 7). The following sections will discuss several of the main approaches that PrOF NMR has been used for in FBDD as well as more detailed case studies.

### The chemical shift perturbation experiment

**Fig. 8:**
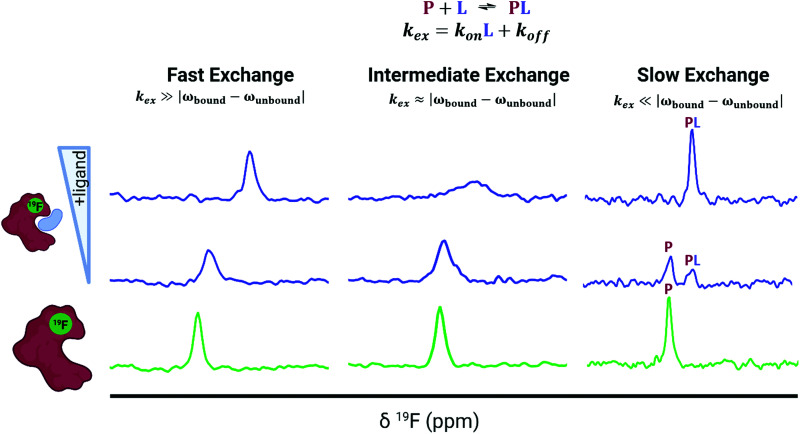
Idealized spectra of protein-observed ^19^F NMR with ligands. Ligands in fast chemical exchange (left), intermediate exchange (middle), and slow exchange indicating the distinct states of free (P) and bound (PL) protein (right).

The predominant method for characterizing protein–ligand interactions *via* PrOF NMR is through a chemical shift perturbation experiment. In such an experiment, the observed resonances are affected differentially depending on the chemical exchange rate which is dictated by the on and off-rates for complex formation and dissociation and the resonance frequency of the bound and unbound states (Fig. 8). For fragment screening, these small molecule interactions tend to be weak (*K*_d_ = mid micromolar to millimolar). Under these conditions a single resonance is observed for the binding interaction, where the chemical shift is the weighted average of the bound and unbound states. Upon ligand titration, the observed change in chemical shift can be fitted to a non-linear regression curve to calculate *K*_d_ using eqn (5).5
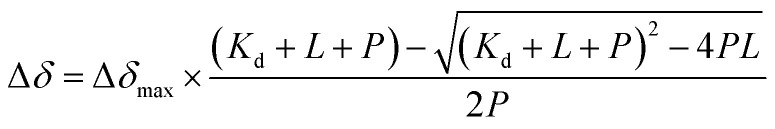


Either a resonance broadening or complete resolution into two distinct resonances indict intermediate and slow exchange binders respectively, and are typically characteristic of higher affinity interactions with slow off-rates.^101^ However, this is not always the case, where even weak ligands can exhibit slow chemical exchange binding.^102^ In chemical shift perturbation experiments, nonspecific effects such as protein aggregation or denaturation can be readily detected which are indicated by a global decrease in resonance intensity or coalescence of protein resonances which helps to identify potential false positives in screens.^41^

### Conformational studies/agonist & antagonist binding case studies with GPCRs

Prior to PrOF NMR fragment-based screening efforts, PrOF NMR was used to study known ligand-protein interactions. One significant example is the study of G protein-coupled receptor (GPCR) signaling using ^19^F NMR. GPCRs make up a large portion of drug targets in FDA approved drugs, but the mechanisms underlying the differing degrees of signaling, or biased signaling, are complex. To study the conformational states of the GPCR β_2_-adrenergic receptor (β_2_AR) upon agonist binding by ^19^F NMR, Liu *et al.* labeled three cysteines on three distinct helices of the intracellular region of β_2_AR with 2,2,2-trifluoroethanethiol (TET).^34^ Cysteine labeling is generally conducted with CF_3_-based tags because the fast internal rotation gives rise to sharp resonances. Cys265 and Cys327 were selected because they are located on helices VI and VII respectively which upon activation are known to change conformations. Cys265 and Cys327 each had two components to their respective ^19^F signal indicating an equilibrium between two different conformations, an inactive and active state. Upon agonist binding which activate G-protein signaling, there was a larger shift towards the active state in helix VI (Cys265) than in helix VII (Cys327). The reverse was observed upon binding of β-arrestin biased ligands, which primarily shifted the equilibrium of helix VII (Cys327) towards an active conformational state. Additionally, the efficacy of partial and full agonists could be distinguished by the relative ratio of the inactive and active resonance, demonstrating PrOF NMR to be a valuable tool in GPCR functional studies. A similar study by Kim *et al.* labeled β_2_AR with a trifluoromethyl tag (–COCF_3_) to study the equilibria of GPCR functional states.^103^ In the apo state, the ^19^F spectra revealed three states (S_1–3_), and upon addition of inverse, partial, and full agonists they were assigned by analyzing the population of each state to two distinct inactive conformers (S_1_ and S_2_) and an intermediate active state (S_3_). When a G-protein mimic was added along with agonist, a fourth state (S_4a_) that was fully active was observed. These studies highlight the sensitivity of PrOF NMR for mapping out binding interactions and conformational landscapes on a therapeutically important class of proteins. PrOF NMR has continued to be used to further characterize the conformational states and allosteric regulation of β_2_AR and the adenosine A_2A_ receptor (A_2A_R).^104–108^ In addition, cysteine labelling ^19^F NMR approaches have also been used to study the dynamics of soluble proteins.^109,110^

### Ligand discovery for the KIX domain: a case study for fragment screening

Although prior studies had used fluorinated proteins to characterize both native and synthetic small molecules, the first use of PrOF NMR as a tool for small-molecule discovery was reported by Pomerantz *et al.* targeting the KIX domain of the coactivator CBP/p300 using a pilot screen of 50 fragments.^35^ Prior NMR work by Wright and Dyson has provided a detailed picture of the molecular mechanisms involving transcriptional activation domains (*e.g.* Myb, CREB, and MLL) binding to two distinct binding sites on this small protein domain.^111,112^ A key insight for this screen focused on the general enrichment of aromatic amino acids at protein–protein interaction interfaces.^113,114^ In the case of KIX, six of the seven aromatic amino acids participated in direct binding interactions and/or being involved in allosteric regulation. PrOF NMR using KIX metabolically labeled with 3-fluorotyrosine (3FY) at five positions covering both binding sites was thus chosen for screening against the KIX domain.

PrOF NMR was attempted to potentially overcome challenges associated with discovering new ligands for dynamic interfaces as previous KIX screening efforts had resulted in only a few inhibitors.^115^ Using fluorine-labeled KIX, the authors characterized the binding and affinity *via* chemical shift perturbation of a known inhibitor of the KIX–CREB protein–protein interaction, napthol–ASE–phosphate,^115^ and fragment molecules 1–10, from a previous tethering fragment screen for the MLL binding site.^116^ Following the success of detecting weak binding ligands, a pilot study of 50 fragments were screened against 3FY–KIX in mixtures of 10 leading to the identification of 1G7, a pyrrole-substituted benzoic acid.^35^

To assess KIX's druggability, the first complete small-molecule screen using PrOF NMR was completed against KIX with an expanded 508-member fragment library by Gee *et al.*^117^ The whole screen was completed in 510 minutes in 85 mixtures (5–6 fragments each) at 40 μM 3FY KIX (20 mg total), demonstrating the rapid nature of PrOF NMR screening as well as the low amount of protein required. Most fragment hits affected the resonance in the MLL binding site (Y631) significantly more than those in the CREB binding site (Y649, Y650, Y658). Further SAR studies identified aryl and phenylacetic acid pharmacophores for the MLL binding site hits, which may mimic hotspot residues in the sequence DIMDFVL found in the KIX-binding MLL peptide. Subsequent studies using more elaborated fragments and a dually labeled KIX protein with 3FY and 4-fluorophenylalanine (4FF) at F612, further elucidated the binding site to be near, but distinct from the MLL binding site.^118^ This result was important as it indicated that the KIX domain could be targeted without competing against endogenous transcription factor interactions.

### Ligand discovery for the SPSB2 and AMA1 proteins: case studies for characterizing binding site interactions

**Fig. 9:**
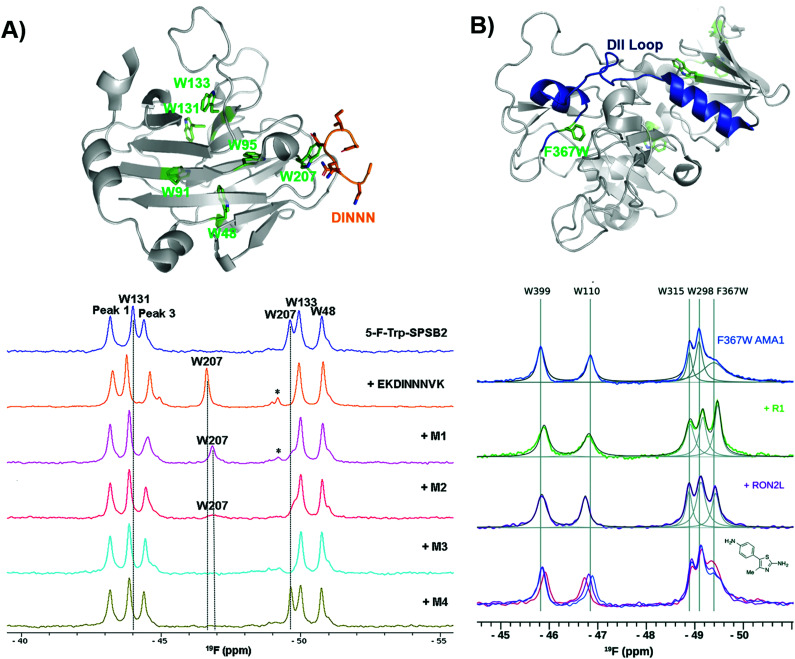
PrOF NMR studies characterizing binding site interactions for SPSB2 and AMA1. (A) SPSB2 (grey) bound to DINNN (orange) with tryptophan residues in green (PDB: 3EMW, top); ^19^F NMR spectra of 5FW-SPSB2 alone, bound to a DINNN peptide, and bound to peptidomimetics M1, M2, M3, M4 (bottom, adapted from ref. 21 with permission from MDPI, copyright 2016). (B) AMA1 in apo form (grey) highlighting the DII loop in blue and tryptophan residues in green, both mutated and native (PDB: 2Z8V, top); ^19^F NMR spectra of F367W 5FW-AMA1 alone, bound to peptides R1 and RON2L, and bound to a series of aminothiazole fragments (0,1,3 μM, bottom, adapted from ref. 126 with permission from Royal Society of Chemistry, copyright 2016).

Another early example of PrOF NMR was described by the Norton lab focusing on the SPRY domain-containing SOCS box protein 2 (SPSB2), a target implicated in infectious diseases for its role in recruiting an E3 ligase to inducible nitrous oxide synthase (iNOS) for degradation.^119^ In this case, PrOF NMR was used as a secondary validation assay. A 5-fluorotryptophan (5FW) construct of SPSB2 was metabolically labeled at six Trp sites, of which W207's resonance was most perturbed upon binding of the N-terminal peptide of iNOS (Fig. 9a). The key peptide residues interacting with the SPRY domain of SPSB2 are DINNN, the indole of W207 is nearby the backbone of an Asn residue of DINNN and solvent exposed under apo conditions explaining why a significant change in chemical shift was observed upon ligand addition. To test the sensitivity for detecting ligand interactions, analogs of hits from a fragment screen (STD and CPMG) were shown to perturb W207 demonstrating their binding in the iNOS binding site. A nonspecific binder known to bind outside the iNOS pocket perturbed resonances that were not W207. An SPBS2 inhibitor was subsequently designed using a stabilized, cyclic peptide analog of DINNN (CP0).^120^ Affinity and kinetics were assessed using SPR and ITC, while PrOF NMR was utilized to confirm that the cyclic peptide bound to the iNOS binding site by perturbation of W207, albeit with ∼0.5 ppm less of a shift in comparison to the linear peptide, indicating a slightly altered binding interaction. Similar studies were done with more stable cyclic analogs, a cystathionine analog and a lactam-bridge analog that were more resistant to REDOX, and perturbed W207 in a similar pattern to CP0.^121^

In subsequent studies using PrOF NMR with a new smaller, more drug-like cyclic pentapeptide, as well as peptidomimetics (M1–M4), the dynamics of binding interactions with SPBS2 were assessed.^122,123^ From these binding studies, it was observed in several cases that W207's resonance sharpened in intensity which suggested binding in the iNOS pocket. This resonance sharpening differed from other ligands, which broaden W207 further suggesting more than one bound conformation (Fig. 9a). To further probe the importance of each residue in the N-termini of iNOS in its interaction with the SPRY domain of SPBS2, PrOF NMR was once again used.^124^ Different extents of broadening were observed in W207, and longer N-termini peptides tended to have higher affinity coupled with less broadening suggesting transient interactions that contribute to a single binding pose.

Similarly, PrOF NMR has been applied to study conformational changes induced upon ligand binding. AMA1, which is found on the surface of parasites, is a target in malarial diseases and disruption of an AMA1–RON2 complex is inhibitory. The domain II (DII) loop of AMA1 is known to be displaced by peptide binders (RON2L and R1); to track loop dynamics upon ligand interaction Ge *et al.* labelled the DII loop with 5FW (F367W, Fig. 9b).^125,126^ Using their fluorinated protein construct, fragments from previous screening efforts were characterized to determine where they bound. One fragment series saw a concentration-dependent sharpening of the F367W resonance with lower magnitude than that of the peptides, suggesting these fragments displace the DII loop but have a lower affinity interaction, while the other two fragment series did not affect F367W's resonance, indicating that they bind in a distinct site that does not induce a change in the DII loop.

### Screening to lead molecules using bromodomains as a case study

**Fig. 10:**
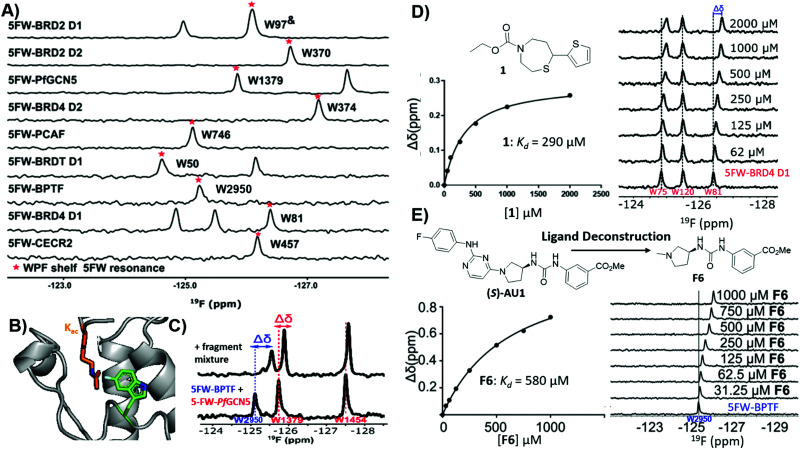
PrOF NMR studies for screening against bromodomains. (A) PrOF NMR spectra of various 5FW-labeled bromodomains. The tryptophan reporter residue located in the WPF shelf denoted by asterisk. (adapted from ref. 131 with permission from MDPI, copyright 2020). (B) The tryptophan reporter residue (5FW) in bromodomains is adjacent to the acetyl lysine (*K*_ac_) binding site. (PDB:3QZS, pending) (C) PrOF NMR titration of a fragment mixture that has hits for both 5FW-BPTF and 5FW *Pf*GCN5 in a dual protein mixture (adapted from ref. 131 with permission from MDPI, copyright 2020). (D) PrOF NMR titration with fragment hit **1** with 5FW-BRD4 D1 and *K*_d_ determination from W81. (adapted from ref. 133 with permission from American Chemical Society, copyright 2019). (E) PrOF NMR titration with deconstructed fragment **F6** (from parent molecule **(S)-AU1**) with 5FW-BPTFand *K*_d_ determination from W2950. (adapted from ref. 136 with permission from Royal Society of Chemistry, copyright 2019).

The utility of PrOF NMR screening in drug discovery has been demonstrated ranging from fragment screening to later stage lead molecule development in targeting the bromodomains of epigenetic regulatory proteins. Bromodomains participate in PPIs with acetylated lysines on histone tails and over half of all 61 bromodomains are enriched with at least four aromatic amino acids in the histone binding site, which can be fluorine labeled.^127^ Furthermore, fluorinated bromodomain constructs have been shown to be sensitive NMR probes for both native ligand and small molecule interactions and have been used to characterize the bromodomains of CBP, CECR2, BPTF, BRD4, BRDT, BRD2, *Pf*GCN5, and PCAF (Fig. 10a and b).^35,127–132^ In a cross-validation study of PrOF NMR, a 930-member fragment library was screened against the first bromodomain of 5FW-labeled BRD4 (BRD4 D1) in mixtures of five using both PrOF NMR and ligand-observed ^1^H CPMG NMR (with and without competitor validation).^41^ Comparing the individual fragments, there was 85% overlap in hit detection when using competitors. PrOF NMR could detect false positives in several cases where a single molecule was found to be the cause of signal reduction in their respective mixtures, possibly through inducing protein aggregation. Both the speed of the PrOF NMR assay and the ability to rank order compounds *via K*_d_ were seen as advantages of PrOF NMR, whereas the removal of a time intensive deconvolution step, lower protein concentration, and use of unlabeled protein are advantages for ^1^H CPMG NMR.

To investigate potential advantages for screening 3D fragments, which have recently come into focus as a way to potentially enhance library diversity,^86,87^ a 467-member 3D-enriched fragment library was screened against 5FW-BRD4 D1 in the same workflow using PrOF and ^1^H CPMG NMR for a direct comparison.^133^ The overall hit rate was lower in comparison to the traditional library screen described above, which is often a concern due to the increased complexity of more 3D fragments. Nonetheless, novel hits were discovered with high affinity and selectivity towards BRD4 D1 over structurally similar bromodomains from the same family (bromodomain and extra-terminal (BET) family (Fig. 10d). Structure–activity relationship studies were conducted on a highly 3D 1,4-acylthiazepane scaffold leading to a ligand efficient (0.41), 20 μM binder (*K*_d_). 1,4-Acylthiazepanes were later shown to have ∼10-fold higher affinity for the second bromodomain of BET proteins, using fluorine-labeled constructs containing the two tandem bromodomains of BRD4 and BRDT.^130^

Bromodomain selectivity is challenging because of their relatively conserved acetyl lysine binding sites. Screening of protein mixtures is one method to enable selectivity information upfront. In PrOF NMR, the simplicity and large chemical shift dispersion of ^19^F resonances allows for ease of screening in mixtures. 5FW-BRD4 D1, which was used in previous screening efforts, and another bromodomain, 5FW-BPTF, have three resonances (W75, W81, W120) and one resonance (W2950) respectively that are sufficiently resolved. As a proof of concept, known inhibitors, including kinase inhibitors selective for BRD4-D1 over BPTF, were shown to selectively perturb W81 (the main reporter resonance in BRD4 D1 located near the binding site) and not W2950 in a mixture.^127^ Urick *et al.* proceeded to screen a 229 small molecule library (published kinase inhibitor set (PKIS I and II)) against 5FW-BRD4 D1 and 5FW-BPTF simultaneously which led to new selective leads for each protein.^129^

Examining the hits for BRD4 D1, SB-284851-BT, a 1,4,5-trisubstituted imidazole, was identified as one of the strongest binders.^129^ When further characterizing selectivity of 1,4,5-trisubstituted imidazoles across BET family bromodomains, Divakaran *et al.* found a preference for binding to the first bromodomains (D1s), with a >55 fold selectivity for BRD4 D1 compared to BRD4 D2 for compound **V**.^134^ Cui *et al.* used this knowledge to design an improved inhibitor, **5**, using the same 1,4,5-trisubstituted imidazole scaffold. Molecule **5** possessed submicromolar affinity and 9–33-fold selectivity for BRD4 D1 over the remaining BET bromodomains.^135^ The selectivity for BRD4-D1 was further confirmed using the fluorine-labeled BRD4 tandem domain construct.^130^

Of the small molecule hits for BPTF from the dual screen with BRD4 D1, the tightest binders were molecules with arylurea motifs.^129^ AU1 was selected for follow-up, which in its racemic form had a *K*_d_ of 2.8 μM by ITC, and by PrOF NMR titrations it was determined that (*S*)-AU1 was the active enantiomer perturbing the reporter resonance, W2950, in the acetyl lysine binding pocket.^129,136^ Furthermore, Kirberger *et al.* used PrOF NMR to guide SAR efforts to improve the solubility and stability concerns, as well as ligand deconstruction to determine the contribution of each fragment to guide potency gains (Fig. 10e).^136^ Unfortunately, there were no significant improvements made in regard to these efforts, but these experiments demonstrate the utility of PrOF NMR in hit optimization. Examining bromodomain selectivity by PrOF NMR, AU1 was shown to be the first selective inhibitor of BPTF with selectivity over BRD4 D1 and moderate selectivity over a highly homologous bromodomain, PCAF. Recently a combined workflow which consists of PrOF NMR fragment screening followed by hit mixture deconvolution by ^1^H CPMG NMR against two bromodomains (BPTF and *Pf*GCN5) simultaneously has been reported, which cuts down on time and resources needed for FBDD (Fig. 10c).^131^ This work on selective bromodomain inhibitor development aided by PrOF NMR highlights ^19^F NMR as a useful tool from screening through chemical probe development.

### Quantification of binding interactions in intermediate and slow exchange

The sensitivity of PrOF NMR is suited for quantifying weak interactions like fragments and peptides, and benefits from increased resolution *versus*^1^H,^15^N-HSQC NMR;^137^ however, ligand binding that is in intermediate chemical exchange can be challenging to quantify. To address this challenge, Stadmiller *et al.* demonstrated the use of ^19^F NMR lineshape analysis to quantify both binding constants and kinetic rates of association of protein–ligand interactions.^138^ The stabilized T22G drosophila drk N-terminal SH3 domain was metabolically labelled with 5FW at W36, located near the binding interface, and the results of 1D ^19^F lineshape analysis were compared to the more well-established 2D ^1^H,^15^N-HSQC NMR. Four reported peptide binders, derived from the SH3–SOS interactions, were evaluated. The peptides exhibited interactions on the intermediate or milliseconds (ms) timescale (exchange rate ∼0.01–100 ms) with SH3, which is appropriate for lineshape broadening analysis. Experimentally derived parameters *K*_d_, *k*_on_, and *k*_off_ were nearly equivalent for all peptides by 1D ^19^F NMR compared to 2D ^1^H,^15^N-HSQC NMR. ^19^F NMR was used here as a faster method, with a five minute acquisition time in comparison to 20 min for 2D HSQC NMR experiments, which along with ease of analysis makes it a useful tool in probing weak ligand interactions.

In some instances weaker binders can exhibit slow exchange. In this case, the integration of the peak intensity of the bound and unbound states can be used to derive a *K*_d_ when the *K*_d_ is near or above the protein concentration avoiding stoichiometric binding. Such a case was observed for the carbohydrate binding protein LecA studied by Shanina *et al.* for lectin inhibitor development.^102^ LecA was metabolically labelled with 5FW at all four native tryptophan residues, of which W42 and W33 are near the binding site of the natural carbohydrate ligand, d-galactose. 5FW-LecA was titrated with known weak binder, *N*-acetyl d-galactosamine. Despite weak affinity, two distinct W42 resonances were detected as ligand was added, indicating slow chemical exchange. The *K*_d_ was calculated from the change in peak intensity of the W42 resonance over ligand concentration and found to be 780 μM. As tryptophans are frequently found in carbohydrate binding sites,^139^ a similar PrOF NMR approach could be applied to lectin drug-discovery campaigns.

### ^19^F NOE and PRE experiments for characterizing biopolymer structure and dynamics

**Fig. 11:**
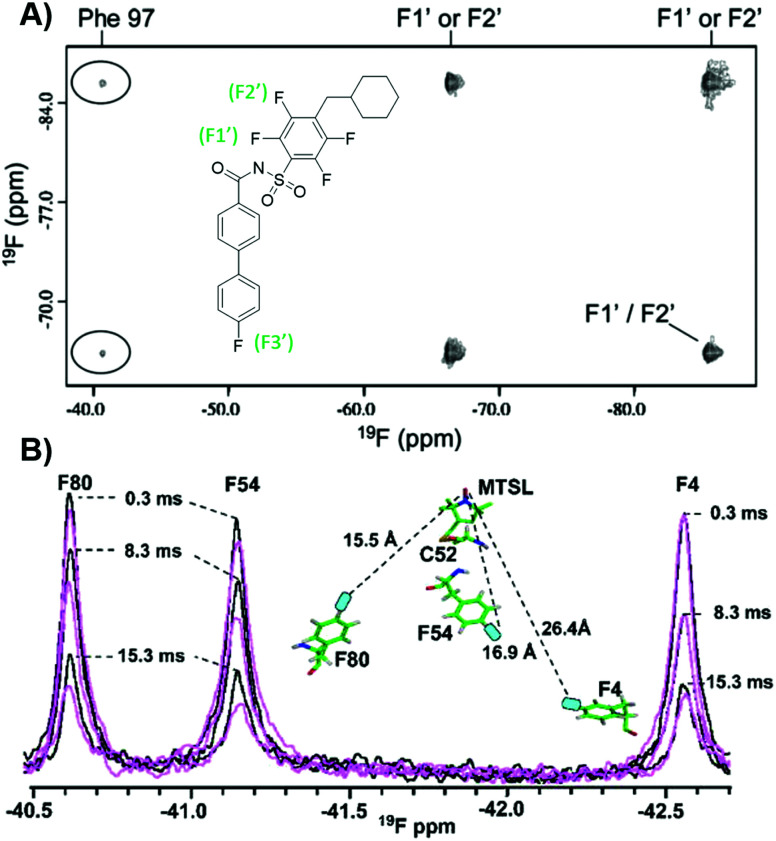
PrOF NMR studies characterizing structure and dynamics of Bcl-xL and lectin CV-N. (A) ^19^F–^19^F NOESY spectra of 4FF-Bcl-xL with a fluorinated ligand, protein–ligand complex crosspeaks circled (adapted from ref. 140 with permission from Springer, copyright 2006). (B) ^19^F NMR spectra of 4FF-MTSL labeled lectin CV-N reduced (black) and oxidized (magenta) at differing relaxation delays (adapted from ref. 146 with permission from Wiley-VCH, copyright 2016).

^19^F NMR can be exploited to obtain structural information of protein–ligand complexes, in particular through extracting interatomic distances. In the case of ^19^F–^19^F NOEs where a protein and ligand are both fluorine-labeled, short range information can be obtained. In the first such intermolecular application with small molecules and proteins, Bcl-xL, a popular anticancer target, was metabolically labelled with 4FF at ten residues.^140^ Addition of a known inhibitor with three fluorine resonances led to observable crosspeaks in the resulting NOESY spectrum (Fig. 11a). This result indicated strong intermolecular NOEs between the ligand and protein consistent with known structural information. Prior work assessing intramolecular interactions has been performed on rhodopsin,^141^ SH3 domains,^142^ and a fatty acid binding protein.^143,144^ More recently intramolecular NOEs were observed between two pentafluorosulfonyl-substituted phenylalanines in a folded protein.^145^

^19^F paramagnetic relaxation enhancement (PRE) experiments complement NOE experiments with longer range information. The Gronenborn lab has showed this technique to give structural information over distances of 12–24 Å, which is comparable to ^1^H PRE (13–25 Å).^146^ This was shown in a case study with lectin cyanovirin-N (CV-N), which was labeled with the commonly used spin label MTSL, introduced by mutated cysteines in a linker region, and differentially fluorine-labeled amino acids, at either W49 or F4, F54, and F80 (Fig. 11b). These type of long distance studies can help elucidate 3D structure when traditional structure studies are not tractable, particularly useful for studies of large, globular proteins, membrane proteins, or fragment-protein complexes which may be more difficult to crystallize. PRE measurements have also been used to show dynamic conformational effects within Myc upon Myc-Max heterodimer formation and interactions with DNA.^147^

### In-cell ^19^F NMR

The absence of ^19^F in biological systems makes the transition to in-cell studies a natural advancement, as it avoids spectral background signals. Early studies were completed in the yeast *S. cerevisiae* by inducible expression of target proteins and labeling with 5FW to measure protein mobility in cells.^148–151^ The scope of this methodology has since been expanded to other systems beyond yeast. An early example in *E. coli* monitored protein conformational changes upon ligand binding to enzymes by site-specific incorporation of trifluoromethyl-l-phenylalanine (tfmF) at TAG nonsense codons.^97^ Seminal contributions in the field by Pielak and co-workers identified methodology to overcome signal broadening of globular proteins in cells. Experiments with ^15^N-labelled and 3FY-labelled globular proteins, like GFP, failed to detect signal due to the high viscosity of cells and nonspecific interactions.^37^ Alternatively, ^19^F NMR spectra for α-synuclein, a disordered protein, both *in vitro* and in cells indicated that the internal motion of disordered proteins could retain resolved ^19^F resonances.^152^ The use of tfmF, which has a CF_3_ group that has its own internal fast bond rotation, also resulted in observable in-cell NMR resonances in two tested globular proteins (GFP and HDH).^37^ In a systematic evaluation of labeling methods for in-cell NMR, it was recommended to first use ^19^F- or ^15^N-labeling, highlighting the power of ^19^F for in cell NMR. The major limitation that still exists is with proteins that interact strongly with other biomolecules in cells, causing signal broadening and making detection difficult.^153^ Despite this, in-cell ^19^F NMR has continued to be used in several applications including protein stability,^154^ protein mobility,^155^ and thermodynamic analyses.^156^ Additionally, novel models systems have been investigated as more robust and less crowded systems for in-cell ^19^F NMR.^157^

## 4. Outlook and future directions

Looking towards the future, we anticipate a sustained increase in innovative new methods for characterizing protein–ligand interactions *via* both ligand-observed and protein-observed ^19^F NMR. Now that significant signal enhancements can be achieved due to the availability of high sensitivity ^19^F NMR cryoprobes, the repertoire of experiments continue to increase, including techniques such as dynamic nuclear polarization.^158^ These experiments significantly augment the structural and quantitative binding information that can be achieved for FBDD. Such advances also continue to fuel innovation in chemical synthesis, as more fluorinated small molecule libraries are available and new synthetic strategies are being developed. In the case of ligand-observed NMR screening, the library sizes for screening has significantly increased and now benefit from improved pulse sequences allowing for larger mixture sizes and higher throughput. We anticipate these methods to continue to be applied to more challenging drug targets such as RNA. ^19^F–^19^F ILOE NMR experiments discussed above can also help researchers address the challenges of fragment linking, even in the absence of crystal structures. New innovations in both ^19^F–^19^F and ^1^H–^19^F NOE experiments are also anticipated in the future to guide inhibitor development.

In our last perspective, we noted the significant challenge of ^19^F CSA as a limitation for protein-observed ^19^F NMR.^19^ However since that time, Aryl ^19^F–^13^C TROSY methods have been reported for biopolymers,^36^ and have now been applied to ligand binding.^50^ However, only a limited set of fluorinated amino acids and nucleic acids have been used, in part due to the requirement of specific ^13^C and ^19^F labeling. Improved access to these building blocks may augment the types of biopolymers which can be studied by this approach. Finally, quantifying the dynamics associated with ligand binding in addition to the thermodynamic binding affinity is another source of continued development in ^19^F NMR. We envision broader adoption of these methods by the research community. While lineshape analysis and PRE measurements were described above, quantitative measurements of slow time scales for binding interactions can also be measured *via* CPMG-based relaxation dispersion ^19^F NMR and newer innovations with off and on resonance R1ρ experiments.^159^ The latter allows for faster dynamics to be assessed while longer time scale protein–ligand interactions can be probed with chemical exchange saturation transfer (CEST) ^19^F NMR.^160,161^ The experiments covered here and those currently being developed will continue to provide a wealth of information for capturing dynamic biomolecular interactions, fueling new advances in drug discovery.

## Author contributions

All authors helped to write and edit the manuscript.

## Conflicts of interest

There are no conflicts of interest to declare.

## Supplementary Material
